# Improvement in Functional Outcomes After Elective Symptomatic Orthopaedic Implant Removal

**DOI:** 10.5435/JAAOSGlobal-D-20-00137

**Published:** 2020-09-01

**Authors:** Benjamin R. Williams, Dylan L. McCreary, Harsh R. Parikh, Melissa S. Albersheim, Brian P. Cunningham

**Affiliations:** From the Department of Orthopaedic Surgery (Dr. Williams, Mr. McCreary, Mr. Parikh, Dr. Albersheim, and Dr. Cunningham), University of Minnesota, Minneapolis, MN; the Department of Orthopaedic Surgery (Mr. Parikh), Regions Hospital, St. Paul, MN; the Department of Orthopaedic Surgery (Dr. Cunningham), Methodist Hospital, St. Louis Park, MN; and TRIA Orthopaedic Center (Dr. Cunningham), Bloomington, MN.

## Abstract

The relative indications for removing symptomatic implants after osseous healing are not fully agreed on. The purpose of this study was to (1) determine whether patients showed improvement in functional outcomes after the removal of symptomatic orthopaedic implants, (2) compare the outcomes between upper and lower extremity implant removal, and (3) determine the rate of implant removal complications.

**Methods:**

A prospective study was conducted between 2013 and 2016. Patients completed a Short Musculoskeletal Function Assessment outcome questionnaire before implant removal and at the 6-month follow-up. Demographic data were stratified and compared between upper and lower extremity groups and between preimplant removal and 6-month postremoval.

**Results:**

Of the 119 patients included in the study, 85 (71.4%) were lower extremity and 34 (28.6%) were upper extremity. Significant improvement after implant removal was seen in the dysfunction index (*P* ≤ 0.001), bother index (*P* ≤ 0.001), and daily activities domain (*P* ≤ 0.001). Depression or anxiety (*P* = 0.016) were statistically significant predictors for an improved Short Musculoskeletal Function Assessment dysfunction index score at 6 months. The complication rate was 10.1% (n=12) for the cohort.

**Discussion:**

Implant removal in both the upper and lower extremity presented notable improvement in dysfunction. Complications that require surgical intervention are extremely rare.

The removal of symptomatic implants after osseous healing is one of the most common orthopaedic procedures performed around the world.^1,2,3,4,5,6^ The exact incidence reported varies based on anatomic location and practice patterns.^7,8,9,10^ Similarly, the recommended indications for implant removal differ between studies. Therefore, no consensus exists on the risks and benefits associated with implant removal.^2,8,11,12,13,14,15,16,17,18,19,20,21,22,23^ Previous studies have shown improvement in pain and discomfort in as low as 53% of patients^22^ and as high as 100% of patients.^17^ Similar variation is seen in complication rates of implant removal with studies reporting a range from 0%^17,21^ to 30%.^19^ Despite the frequency of the procedure, and the inconsistent evidence of the risks and benefits, limited prospective outcome data are available to guide treatment decisions.^16,24^

The primary purpose of this study was to determine whether patients had improved functional outcomes after the removal of symptomatic orthopaedic implants after osseous union. The primary outcome was the change in the Short Musculoskeletal Function Assessment (SMFA) patient-reported outcome (PRO) questionnaire. Secondary purposes included comparing the outcomes of implant removal in the upper extremity to that of the lower extremity and determining the perioperative compilation rates after implant removal. The study hypothesized that the removal of symptomatic orthopaedic implants would result in improved patient function measured through improved PROs.

## Methods

### Patients

After Institutional Review Board approval, a prospective observational study assessing outcomes after the removal of symptomatic orthopaedic implants was conducted at two academic institutions (a level 1 trauma center and a surgical center) between 2013 and 2016. The indications for implant removal were aseptic symptomatic implants after bony union. Symptomatic implants were defined as generalized discomfort or point tenderness over the implant. Inclusion criteria for the study were (1) age older than or equal to 18 years old, (2) initial closed injury if a fracture, (3) absence of baseline peripheral neuropathy, and (4) a complete SMFA PRO questionnaire before implant removal. Patients were excluded if they were found to have (1) previous or current infection at the surgical site or of the underlying implant, (2) osseous nonunion, or (3) polytrauma patients. A follow-up outcome questionnaire was completed at 6 months after implant removal. Baseline demographics and outcome scores were compared between the cohort of patients who returned for the follow-up at 6 months and the cohort of patients that was lost to follow-up to assess for any differences. Demographic characteristics were collected and involved age, sex, body mass index (BMI), workman's compensation, a 10-year history of smoking, positive clinical previous diagnosis of depression or anxiety disorders, the American Society of Anesthesiologists Physical Status classification system score, and the time duration of the implant from the primary surgery to the removal surgery.

### Outcomes

The SMFA is a patient-reported standardized musculoskeletal functionality outcome questionnaire.^25^ This is a globally implemented and validated outcome instrument used to evaluate a wide range of musculoskeletal disorders.^26,27^ Because the SMFA is a general lower and upper extremity outcome instrument, it allows for the removal of symptomatic implant to be evaluated within the broad context of musculoskeletal conditions. The scale ranges from 0 to 100, with 0 representing the least dysfunction and 100 representing complete dysfunction. A negative change in the score from baseline to 6 months represents an improvement in the overall function. The outcome questionnaire was completed just before their 6-month follow-up by mail (and brought in to clinic) or in person at their 6-month follow-up visit. The SMFA outcome scores in this study were evaluated across the dysfunction and bother indices and the daily activities domain. The baseline and 6-month follow-up SMFA scores were evaluated and compared within the overall sample and within upper and lower extremity cohort populations. Intraoperative and postoperative complications were collected from the patient's surgical report and follow-up clinic notes. A complication was considered any infection (deep or superficial), nerve or artery injury, wound healing problems, or any reason for a return to the operating room.

### Statistical Analysis

Descriptive statistics were used to evaluate the demographic data. Demographic characteristics involved age, sex, BMI, workman's compensation, a 10-year history of smoking, positive clinical previous diagnosis of depression or anxiety disorders, the American Society of Anesthesiologists Physical Status classification system score, and the time duration of the implant from the primary surgery to the removal surgery. Demographic data were stratified and compared between the upper and lower extremity groups using a combination of Wilcoxon rank-sum tests and chi-squared tests. The differences between baseline preimplant removal and 6-month SMFA scores were compared using nonparametric Wilcoxon signed-rank tests within the overall sample and both upper and lower extremity study populations. A logistic relative risk (RR) regression analysis was conducted to assess for potential predictors of an increased SMFA dysfunction index score at the follow-up, respective to the subject's original baseline score.

Descriptive data are summarized as mean, standard deviation, and the 95% confidence interval. The level of statistical significance was set at *P* ≤ 0.05. Statistical analyses were performed with SAS 9.4 (SAS Institute, Cary, NC).

## Results

The inclusion and exclusion criteria were met by 279 patients who completed a preimplant removal baseline outcome questionnaire. Of those, 119 (42.7%) returned at 6 months and completed the follow-up outcome questionnaire. The baseline demographics between the study cohort and the group that that was lost to follow-up were comparable, except for age (*P* < 0.001) and sex (*P* = 0.014) (Table 1). Furthermore, the baseline SMFA scores were similar between the study group and the group that that was lost to follow-up (functional: *P* = 0.324; daily activities domain: *P* = 0.568; Bother: *P* = 0.911) (Table 1).

**Table 1:** Comparing Population Characteristics Between Sample and Participants Who Did Not Return for the 6-month Follow-up

Factor	Study Sample (n = 119)	Lost to Follow-up (n = 160)	*P* Value
Sex, n (%)			**0.014**^b^
Female	75 (63.0)	74 (46.4)	
Male	44 (37.0)	86 (53.6)	
Age	49.0 ± 16.7 (14.8, 19.1)	38.7 ± 14.6 (35.7, 41.6)	**<0.001**^c^
BMI	27.1 ± 5.5 (26.1, 28.1)	28.1 ± 5.2 (27.1, 29.1)	0.16^c^
Workman compensation, n (%)			0.132^b^
No	108 (90.8)	156 (95.6)	
Yes	11 (9.2)	7 (4.4)	
Smoking history, n (%)			0.710^b^
No	81 (68.1)	113 (70.6)	
Yes	38 (31.9)	47 (29.4)	
Depression or anxiety,^a^ n (%)			0.273^b^
No	77 (64.7)	18 (73.8)	
Yes	42 (35.3)	42 (26.2)	
Injured extremity, n (%)			0.088^b^
Lower extremity	85 (71.4)	98 (61.3)	
Upper extremity	34 (28.6)	62 (38.7)	
Implant duration time (mo)	22.3 ± 32.3 (16.4, 28.2)	30.9 ± 46.8 (21.5, 40.3)	0.128^c^
American Society of Anesthesiologists Physical Health classification, n (%)			0.150^b^
I	65 (54.6)	105 (65.6)	
II	43 (36.1)	42 (26.3)	
III	11 (9.3)	13 (8.1)	
Functional	12.0 ± 10.9 (10.1-13.9)	11.1 ± 10.9 (9.4-12.8)	0.324^d^
Daily activities domain	11.6 ± 12.2 (9.4-13.8)	11.2 ± 13.3 (9.1-13.3)	0.568^d^
Bother	13.8 ± 12.7 (11.3-16.3)	13.5 ± 12.6 (11.4-15.6)	0.911^d^

BMI = body mass index

a Depression or anxiety was confirmed via either a positive clinical diagnosis or prescribed medications for these disorders.

b Resulting *P*-value for a chi-square test between procedural groups.

c Resulting *P*-value for a two-sample *t*-test.

d Resulting *P*-value of a Wilcoxon rank-sum test between procedural groups because a Shapiro-Wilk test confirms a non-normal distribution (*P* < 0.05).

A summary of the baseline study sample and lost to follow-up sample characteristics. Significance for bolded entries is *p* < 0.05.

Of the 119 patients included in the study, 85 (71.4%) were lower extremity and 34 (28.6%) were upper extremity. Between upper extremity and lower extremity subgroups, no significant differences were identified in the demographic variables, nor the baseline SMFA scores (Table 2). The upper extremity sites for implant removal were olecranon (49%), clavicle (29%), distal radius (9.8%), ulnar shaft (9.8%), and radius and ulnar shaft (2.4%). The lower extremity sites for implant removal were ankle (47%), metatarsal and tarsal bones (33%), tibial shaft (6.7%), patella (5.9%), tibial plateau (5.9%), and distal femur (1.7%).

**Table 2:** Population Characteristics for Sample Population Between 2013 and 2016, Stratified by Injury Type (N = 119)

Factor	Lower Extremity (n = 85)	Upper Extremity (n = 34)	*P* Value
Sex, n (%)			0.063^b^
Female	58 (68.2)	17 (50.0)	
Male	27 (31.8)	17 (50.0)	
Age	47.5 ± 15.7 (44.1 to 50.9)	52.7 ± 18.6 (46.2 to 59.1)	0.129^c^
BMI	27.3 ± 5.0 (26.2 to 28.4)	26.5 ± 6.5 (24.3 to 28.8)	0.551^c^
Workman compensation, n (%)			0.414^b^
No	75 (89.3)	32 (94.1)	
Yes	9 (10.7)	2 (5.9)	
Smoking history, n (%)			**0.025**^b^
No	63 (74.1)	18 (52.9)	
Yes	22 (25.9)	16 (47.1)	
Depression or anxiety,^a^ n (%)			0.396^c^
No	57 (67.1)	20 (58.8)	
Yes	28 (32.9)	14 (41.2)	
Implant duration time (mo)	29.0 ± 45.5 (22.8 to 35.2)	11.2 ± 7.9 (8.5 to 14.0)	**<0.001**^c^
SMFA follow-up time (mo)	5.8 ± 0.9 (5.6 to 6.0)	5.9 ± 0.7 (5.6 to 6.1)	0.681^c^
American Society of Anesthesiologists Physical Health classification, n (%)			0.984^b^
I	46 (54.1)	19 (55.9)	
II	31 (36.5)	12 (35.3)	
III	8 (9.4)	3 (8.8)	
∆Functional	−4.1 ± 9.0 (−6.0 to −2.1)	−2.5 ± 5.8 (−4.5 to −0.4)	0.244^d^
∆Bother score	−2.0 ± 13.6 (−5.0 to 0.9)	−3.5 ± 9.4 (−7.1 to −0.1)	0.940^d^
∆Daily activities domain	−4.8 ± 11.1 (−7.3 to −2.4)	−3.7 ± 9.2 (−6.9 to −0.5)	0.213^d^

BMI = body mass index; SMAFA = Short Musculoskeletal Function Assessment

a Depression or anxiety was confirmed via either a positive clinical diagnosis or prescribed medications for these disorders.

b Resulting *P*-value for a chi-square test between procedural groups.

c Resulting *P*-value for a two-sample *t*-test.

d Resulting *P*-value of a Wilcoxon ranked-sum test between procedural groups because a Shapiro-Wilk test confirms a non-normal distribution (*P* < 0.05).

A summary of study sample characteristics. Significance for bolded entries is *p* < 0.05.

**Figure 1:**
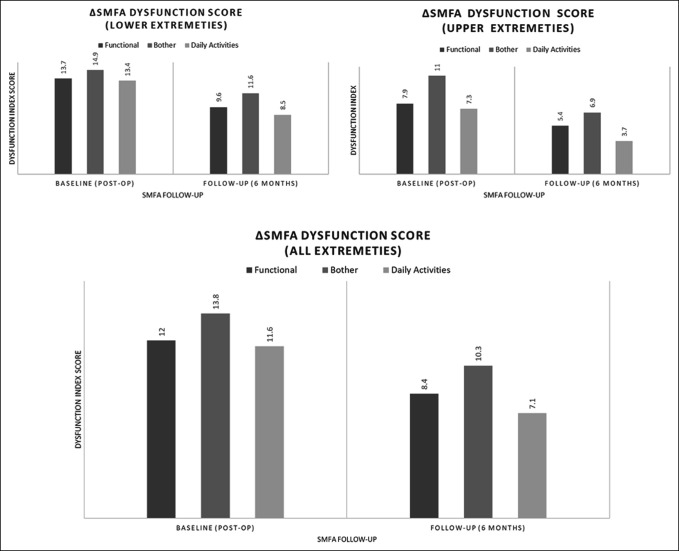
Chart showing the Mean Short Musculoskeletal Functional Assessment (SMFA) index scores measured at both follow-up periods postsurgery. Graphs represent the summaries for all injury types and stratified for injury type, lower extremities, and upper extremities. The SMFA measures afflicted area dysfunction, 0-100. A value of 0 represents the best functional outcome with 100 representing the worst functional outcome. Therefore, a decreasing SMFA-index score represents an improved functionality to the operated region.

Of the study participants, the mean age was 49.0 ± 16.7 [46.0, 52.0] years, 75 (63.0%) were women, and the mean BMI was 27.1 ± 5.5 [26.1, 28.1]. The mean time from primary surgery to implant removal was 22.3 ± 32.3 [16.4, 28.2] months (Table 1).The mean preimplant removal baseline SMFA scores for the overall study population were 12.0 ± 11.2 [10.3, 13.8] for the dysfunction index, 13.8 ± 15.3 [11.4, 16.2] for the bother index, and 11.6 ± 13.1 [9.6, 13.7] for the daily activities domain. The 6-month follow-up SMFA scores for the study group were 8.4 ± 11.3 [6.8, 10.8] for the dysfunction index, 10.3 ± 13.7 [8.1, 12.4] for bother index, and 7.1 ± 13.5 [4.9, 9.2] for daily activities domain (Figure 1).

The mean difference in the SMFA scores, from baseline to 6 months after implant removal, were statistically significant across all indices for the overall study population and for the extremity subgroups (Table 3). Furthermore, the mean change in outcome scores was similar between the upper extremity and lower extremity groups (Table 2). The overall study groups' mean SMFA score change was −3.6 ± 8.2 [−5.1, −2.1] for the dysfunction index, −2.4 ± 12.6 [−4.8, −0.1] for the bother index, and −4.5 ± 10.6 [−6.4, −2.5] for the daily activities domain. A total of 85 study participants reported an improved SMFA dysfunction index score, 60 of 85 (70.6%) lower extremity participants, and 25 of 34 (73.5%) upper extremity participants.

**Table 3:** Change in Reported Outcome Scores for Study Sample

Factor	Change (∆) ± SD (95% CI)	*P* Value^a^
∆Functional	−3.6 ± 8.2 (−5.1 to −2.1)	**<0.001**
∆Bother score	−2.4 ± 12.6 (−4.8 to −0.1)	**<0.001**
∆Daily activities domain	−4.5 ± 10.6 (−6.4 to −2.5)	**<0.001**
Lower extremity procedures (n = 85)		
∆Functional	−4.1 ± 9.0 (−6.0 to −2.1)	**<0.001**
∆Bother score	−2.0 ± 13.6 (−5.0 to 0.9)	**<0.001**
∆Daily activities domain	−4.8 ± 11.1 (−7.3 to −2.4)	**<0.001**
Upper extremity procedures (n = 34)		
∆Functional	−2.5 ± 5.8 (−4.5 to −0.4)	**0.034**
∆Bother score	−3.5 ± 9.4 (−7.1 to −0.1)	**0.021**
∆Daily activities domain	−3.7 ± 9.2 (−6.9 to −0.5)	**0.047**

CI = confidence interval; SMFA = Short Musculoskeletal Function Assessment

a Shapiro-Wilk tests for all three change outcomes were significant, confirming non-normality. Therefore, all outcome measures were tested for significance by a nonparametric Wilcoxon signed-rank test.

Summary of the mean change in outcomes values between baseline and after a minimum 5-month follow-up period. Outcome change values were only derived for participants who completed both the baseline and follow-up SMFA surveys. Significance for bolded entries is *p* < 0.05.

The logistic RR regression analysis identified only previous diagnosis of depression or anxiety (RR = 1.36 [1.06, 1.74]; *P* = 0.016) as statistically significant predictors for an improved SMFA dysfunction index score at 6 months (Table 4).

**Table 4:** Relative Risk (RR) Estimates for Covariate Associations With Binary Change in Short Musculoskeletal Function Assessment (SMFA) Functional Index Scores With RR Regression

Covariate	RR (95% CI)	Wald χ2	*P* Value
Extremity^a^	1.05 (0.82-1.33)	0.11	0.743
Age	1.00 (0.99-1.00)	0.17	0.684
Sex^b^	0.84 (0.65-1.08)	1.83	0.176
BMI	0.99 (0.98-1.01)	0.37	0.543
Workman compensation	1.02 (0.70-1.50)	0.01	0.903
10-yr smoking history	0.99 (0.78-1.27)	0.00	0.951
ASA	0.94 (0.78-1.12)	0.51	0.474
Depression or anxiety^c^	1.36 (1.06-1.74)	**5.76**	**0.016**

ASA = American Society of Anesthesiologists; BMI = body mass index; CI = confidence interval

a The extremity variable was assessed using the lower extremity group as the coded reference.

b Gender variable was assessed using the female group as the coded reference.

c Significance is *p* < 0.05.

Logistic RR regression to assess for covariate influences to the improvement in SMFA functional index outcome. The regression assesses for the probability of having an improved SMFA functional score outcome at the follow-up. Significance for bolded entries is p < 0.05.

Among the 119 patients who met the inclusion and exclusion criteria and completed a baseline and 6-month follow-up SMFA survey, 12 patients (10.1%) had a perioperative complication of which 1 patient (0.8%) required a return trip to the operating room. In the lower extremity subgroup, 3 patients (3.4%) had a superficial skin infection, 4 cases (1.4%) resulted in broken implants requiring broken implant removal sets and increased surgical time, and one patient developed a sural nerve neurapraxia that was still present at the 6-month time point. In the upper extremity subgroup, 2 patients (0.7%) had a superficial skin infection, 1 case (0.4%) resulted in broken implants requiring broken implant removal sets and increased operative time, and 1 patient (0.4%) developed a median nerve neurapraxia that resolved by the 6-month time point. All but one of the infections resolved with oral antibiotics. One (0.7%) patient required a return trip to the operating room for a wound irrigation and débridement. This was an overall infection rate of 4.2% for the upper and lower extremity patients.

## Discussion

Removal of implants, after osseous healing, is one of the most common procedures performed in orthopaedics; however, no consensus exists on the indications, risks, or benefits of the procedure, and little prospective outcomes research exists to support current practices.^1,2,7,8,9,10,12,16^ The purpose of this study was to determine whether patients who had symptomatic implants removed would show improved functional outcomes. This study found a statistically significant improvement in the SMFA dysfunction index (*P* ≤ 0.001), bother index (*P* ≤ 0.011), and daily activities domains (*P* ≤ 0.001) at 6 months after implant removal. No statistical difference was seen in the SMFA scores for patients undergoing lower extremity implant removals versus upper extremity implant removals, although both reached statistical significance independently.

Many surgeons cite unexplained pain or discomfort at the site of retained orthopaedic implants as an indication for implant removal.^2,12,13,16,28^ Most of the previous studies assessing the outcomes after implant removal used the level of discomfort or pain as the primary outcome measure, with most administering a nonvalidated pain tool and/or a visual analog scale.^18,20,22,29,30^ Studies assessing the changes in discomfort or pain found improvements in 53% to 74% of patients after implant removal.^22,29,30^ When administering the validated visual analog scale to measure pain before and after implant removal, 73% to 76% of patients reported an improvement in pain.^17,20^

Few previous studies have assessed functional outcomes after orthopaedic implant removal.^17,22^ A study that administered a retrospective nonvalidated survey to 332 patients found an improvement in 55% of patients with implant removals.^22^ A prospective study by Minkowitz et al,^17^ using the SMFA as the primary outcome, assessed functional outcomes in 57 patients after implant removal after osseous healing for fracture fixation. The authors reported that all patients in the study had functional improvement by the end of the follow-up period. Our study had similar results, demonstrating improvement in the dysfunction and bother indices and daily activity domain of the SMFA in 88 (74%) of patients at the end of the study period. Minkowitz et al found a greater improvement in SMFA scores for the lower extremity compared with the upper extremity. Similarly, our study had a larger cohort of lower extremity patients; however, no notable difference was observed between patients with upper versus lower extremity implants removed. The soft tissue coverage of the upper extremity may offer an explanation for this difference because the clavicle and olecranon are prominent structures for implants with limited subcutaneous tissue coverage, regardless of body habitus. Importantly, the study by Minkowitz et al had an upper extremity cohort of seven patients, which may limit statistical comparison. Further analysis with a larger study population is necessary to determine the difference between anatomical locations.

After implant placement, the forces experienced by bone and within a joint are altered, which may cause the implants to be symptomatic.^24,31-33^ Another important factor to address in elective implant removal is the cost and economic burden. There are very few studies that have assessed the cost of implant removal outside the setting of infection.^5,34^ A pediatric database study found that the cost of inpatient implant removal to be on average $11,792.^34^ However, this included implant removals for infections. A recent study reviewed the cost of implant removal for ankle fractures.^5^ The authors reviewed 185 patients who underwent implant removal in Ireland and found the cost to be on average $1,367. Further prospective studies are needed to assess the economic burden of implant removal surgeries.

Patient comorbidities may be predictors for improvement in discomfort and function after implant removal. The previous study by Minkowitz et al^17^ found that implant removal in older patients predicted greater functional improvement. Our study did not find this same conclusion because age showed no relation to functional improvement. However, our study did identify that a previous diagnosis of depression or anxiety had a statistically significant relation to improvement in the SMFA dysfunctional index after implant removal. Previous literature has consistently shown that patients with active depression or anxiety have higher levels of pain, greater expectations, and poorer outcomes than those who do not.^35,36,37,38^ Interpretation of these results is difficult. It may be that some patients are more aware of their retained implants. A study with a larger cohort specifically assessing the association with mental health is needed to understand this finding.

A survey of orthopaedic surgeons in 2008 found that 48% felt that implant removal was riskier than leaving the retained implant in place.^28^ The complication rate of implant removal has been reported to be as low as 0% and as high as 30%.^8,12,19^ The most commonly reported complication in multiple studies is superficial infections.^19,20,22^ Our study found a complication rate of 10.1% (n = 12/119), of which 4.2% (n = 5/119) were superficial infections that resolved with antibiotics. Lower perioperative complication rates seem to be reflected in more recent literature. Advancement in implants and removal techniques likely contribute to the recent decline in complication rates. A large case series of 1,545 patients who underwent elective implant removal from 2009 to 2012 found an overall complication rate of 5.1%, with calcaneal implant removal demonstrating the highest complication rate.^11^ Our study's findings are consistent with the current literature.

There were several limitations to this study. The study lacked a control group who met the inclusion criteria but did not undergo implant removal. This weakness could be best addressed with a randomized trial where patients who refuse to be randomized are followed with the same outcome measures and analyses performed both for “as-randomized” and “as-treated” patient groups. However, this study was prospective and used a validated patient-reported outcome measure. The SMFA outcome score is a popular patient-reported outcome tool commonly used to study a broad range of musculoskeletal disorders.^39,40^ Several of the previous studies assessed only changes in pain or discomfort or used nonvalidated measures of function.^18,20,22,29,30^ An additional limitation is that the anatomic locations of implant removal groups were too small for intergroup analysis. A future study with greater group size powered to assess the differences between anatomic locations of implant removal is needed to address this limitation. In addition, the group sizes between the extremity groups are markedly different, with the lower extremity group size (n = 85) being nearly three times the size of the upper extremity group (n = 34). Higher reported rates of lower extremity implant removal are consistent with previous studies, possibly indicating implant removal is more common in the lower extremity.^11,17,22^ Future studies could focus on creating a better distribution between the two cohort sizes to ensure that the statistical results are not driven by cohort sizes. Our study found an association between a previous diagnosis of anxiety or depression and improved functional outcomes. We did not use a mental health patient-reported outcome tool or assess the patient's level of anxiety or depression at the clinic visits. The diagnosis was captured in patients' electronic medical record, and therefore, this association should be used as a stepping stone for further studies on interactions between mental health and outcomes in orthopaedic care.

Implant removal in both the upper and lower extremity allows statistically significant improvement in dysfunction; the clinical significance of this improvement, however, is not known. These results would be best corroborated through a randomized trial where patients who refuse to be randomized are followed with the same outcome measures and analyses performed both for “as-randomized” and “as-treated” patient groups. In addition, greater cohort sizes are needed to evaluate the effects of anatomic location and patient comorbidities.

## References

[1] Rutkow IM: Orthopaedic operations in the United States, 1979 through 1983. J Bone Joint Surg Am 1986;68-A:716-719. 3722227

[2] Bostman O Pihlajamaki H: Routine implant removal after fracture surgery: A potentially reducible consumer of hospital resources in trauma units. J Trauma 1996;41:846-849. 8913214 10.1097/00005373-199611000-00013

[3] Lee C Feaker DA Ostrofe AA Smith CS: No difference in risk of implant removal between orthogonal mini-fragment and single small-fragment plating of midshaft clavicle fractures in a military population: A preliminary study. Clin Orthop Relat Res 2020;478:741-749. 32229745 10.1097/CORR.0000000000000877 PMC7282585

[4] Ellwein A Lill H Warnhoff M: Can low-profile double-plate osteosynthesis for olecranon fractures reduce implant removal? A retrospective multicenter study. J Shoulder Elbow Surg 2020;29:1275-1281. 32284307 10.1016/j.jse.2020.01.091

[5] Fenelon C Murphy EP Galbraith JG Kearns SR: The burden of hardware removal in ankle fractures: How common is it, why do we do it and what is the cost? A ten-year review. Foot Ankle Surg 2019;25:546-549. 30321944 10.1016/j.fas.2018.05.006

[6] Cronin PK Watkins IT Riedel M Kaiser PB Kwon JY: Implant removal matrix for the upper extremity orthopedic surgeon. Arch Bone Jt Surg 2020;8:99-111. 32090153 10.22038/abjs.2019.36525.1962 PMC7007717

[7] Lovald S Mercer D Hanson J: Complications and hardware removal after open reduction and internal fixation of humeral fractures. J Trauma 2011;70:1273-1277. 21610440 10.1097/TA.0b013e318215bedd

[8] Naumann MG Sigurdsen U Utvåg SE Stavem K: Incidence and risk factors for removal of an internal fixation following surgery for ankle fracture: A retrospective cohort study of 997 patients. Injury 2017;47:1783-1788. 10.1016/j.injury.2016.05.011 27262772

[9] Lutsky KF Beredjiklian PK Hioe S Bilello J Kim N Matzon JL: Incidence of hardware removal following volar plate fixation of distal radius fracture. J Hand Surg Am 2015;40:2410-2415. 26527594 10.1016/j.jhsa.2015.09.017

[10] Ashman BD Slobogean GP Stone TB: Reoperation following open reduction and plate fixation of displaced mid-shaft clavicle fractures. Injury 2014;45:1549-1553. 24893919 10.1016/j.injury.2014.04.032

[11] Suda AJ Heilgeist E Tinelli M Bischel OE: High early post-operative complication rate after elective aseptic orthopedic implant removal of upper and lower limb. J Orthop Res 2018;36:1035-1039. 28862357 10.1002/jor.23718

[12] Vos D Verhofstad MHJ: Indications for implant removal after fracture healing: A review of the literature. Eur J Trauma Emerg Surg 2013;39:327-337. 26815392 10.1007/s00068-013-0283-5

[13] Jamil W Allami M Choudhury MZ Mann C Bagga T Roberts A: Do orthopaedic surgeons need a policy on the removal of metalwork? A descriptive national survey of practicing surgeons in the United Kingdom. Injury 2008;39:362-367. 18242607 10.1016/j.injury.2007.10.028

[14] Wang S Saha R Shah N: Effect of intravenous acetaminophen on postoperative opioid use in bariatric surgery patients. Pharmacol Ther 2015;40:847-850. PMC4671470 26681907

[15] Barcak EA Beebe MJ Weinlein JC: The role of implant removal in orthopedic trauma. Orthop Clin North Am 2018;49:45-53. 29145983 10.1016/j.ocl.2017.08.014

[16] Busam ML Esther RJ Obremskey WT: Hardware removal: Indications and expectations. J Am Acad Orthop Surg 2006;14:113-120. 16467186 10.5435/00124635-200602000-00006

[17] Minkowitz RB Walsh M Egol KA Bhadsavle S: Removal of painful orthopaedic implants after fracture union. J Bone Joint Surg 2007;89-A:1906-1912. 10.2106/JBJS.F.01536 17768185

[18] Brown OL Dirschl DR Obremskey WT: Incidence of hardware-related pain and its effect on functional outcomes after open reduction and internal fixation of ankle fractures. J Orthop Trauma 2001;15:271-274. 11371792 10.1097/00005131-200105000-00006

[19] Sanderson PL Ryan W Turner PG: Complications of metalwork removal. Injury 1992;23:29-30. 1541495 10.1016/0020-1383(92)90121-8

[20] Williams AA Witten DM Duester R Chou LB: The benefits of implant removal from the foot and ankle. J Bone Joint Surg 2012;94-A:1316-1320. 10.2106/JBJS.J.01756 22810403

[21] Boyle MJ Gao R Frampton CMA Coleman B: Removal of the syndesmotic screw after the surgical treatment of a fracture of the ankle in adult patients does not affect one-year outcomes: A randomised controlled trial. Bone Joint J 2014;96B:1699-1705. 10.1302/0301-620X.96B12.34258 25452376

[22] Reith G Schmitz-Greven V Hensel KO: Metal implant removal: Benefits and drawbacks—A patient survey. BMC Surg 2015;15:1-8. 26250649 10.1186/s12893-015-0081-6 PMC4528685

[23] Gyuricza C Carlson MG Weiland AJ Wolfe SW Hotchkiss RN Daluiski A: Removal of locked volar plates after distal radius fractures. J Hand Surg Am 2011;36:982-985. 21571444 10.1016/j.jhsa.2011.03.032

[24] Vos D Hanson B Verhofstad M: Implant removal of osteosynthesis: The Dutch practice. Results of a survey. J Trauma Manag Outcomes 2012;6:6. 22863279 10.1186/1752-2897-6-6 PMC3485133

[25] Barei DP Agel J Swiontkowski MF: Current utilization, interpretation, and recommendations: The musculoskeletal function assessments (MFA/SMFA). J Orthop Trauma 2007;21:738-742. 17986893 10.1097/BOT.0b013e31815bb30f

[26] Swiontkowski MF Engelberg R Martin DP Agel J: Short musculoskeletal function assessment questionnaire: Validity, reliability, and responsiveness. J Bone Joint Surg Am 1999;81-A:1245-1260. 10.2106/00004623-199909000-00006 10505521

[27] Lindahl M Andersen S Joergensen A Frandsen C Jensen L Benedikz E: Cross-cultural adaptation and validation of the Danish version of the Short Musculoskeletal Function Assessment questionnaire (SMFA). Qual Life Res 2017;27:1-5. 10.1007/s11136-017-1643-0 28677076

[28] Hanson B van der Werken C Stengel D: Surgeons' beliefs and perceptions about removal of orthopaedic implants. BMC Musculoskelet Disord 2008;9:73. 18501014 10.1186/1471-2474-9-73 PMC2430567

[29] Jung HG Kim JI Park JY Park JT Eom JS Lee DO: Is hardware removal recommended after ankle fracture repair? Biomed Res Int 2016;2016:1-7. 10.1155/2016/5250672 PMC5081426 27819005

[30] Jacobsen S Honnens de Lichtenberg M Jensen CM Torholm C: Removal of internal fixation—The effect on patients' complaints: A study of 66 cases of removal of internal fixation after malleolar fractures. Foot Ankle Int 1994;15:170-171. 7951948 10.1177/107110079401500402

[31] Samiezadeh S Avval PT Fawaz Z Bougherara H: Biomechanical assessment of composite versus metallic intramedullary nailing system in femoral shaft fractures: A finite element study. Clin Biomech 2014;29:803-810. 10.1016/j.clinbiomech.2014.05.010 24951320

[32] Disegi JA Eschbach L: Stainless steel in bone surgery. Injury 2000;31(suppl 4):2-6. 10.1016/s0020-1383(00)80015-7 11270076

[33] Cheung G Zalal P Spelt JK Papini M: Finite element analysis of a femoral retrograde intramedullary nail subject to gait loading. Med Eng Phys 2004;26:93-108. 15036177 10.1016/j.medengphy.2003.10.006

[34] Boulos A DeFroda SF Kleiner JE Thomas N Gil JA Cruz AI: Inpatient orthopaedic hardware removal in children: A cross-sectional study. J Clin Orthop Trauma 2017;8:270-275. 28951646 10.1016/j.jcot.2017.06.020 PMC5605744

[35] Dekker AP Salar O Karuppiah SV Bayley E Kurian J: Anxiety and depression predict poor outcomes in arthroscopic subacromial decompression. J Shoulder Elbow Surg 2016;25:873-880. 27068379 10.1016/j.jse.2016.01.031

[36] Ali A Lindstrand A Sundberg M Flivik G: Preoperative anxiety and depression correlate with dissatisfaction after total knee arthroplasty: A prospective longitudinal cohort study of 186 patients, with 4-year follow-up. J Arthroplasty 2017;32:767-770. 27692782 10.1016/j.arth.2016.08.033

[37] Menendez ME Baker DK Oladeji LO: Psychological distress is associated with greater perceived disability and pain in patients presenting to a shoulder clinic. J Bone Joint Surg Am 2015;97:1999-2003. 26677233 10.2106/JBJS.O.00387

[38] Cody EA Mancuso CA Burket JC Marinescu A MacMahon A Ellis SJ: Patient factors associated with higher expectations from foot and ankle surgery. Foot Ankle Int 2017;38:472-478. 28196438 10.1177/1071100717690807

[39] Dattani R Slobogean GP O'Brien PJ: Psychometric analysis of measuring functional outcomes in tibial plateau fractures using the Short Form 36 (SF-36), Short Musculoskeletal Function Assessment (SMFA) and the Western Ontario McMaster Osteoarthritis (WOMAC) questionnaires. Injury 2013;44:825-829. 23246562 10.1016/j.injury.2012.10.020

[40] Bouffard J Bertrand-Charette M Roy JS: Psychometric properties of the Musculoskeletal Function Assessment and the Short Musculoskeletal Function Assessment: A systematic review. Clin Rehabil 2016;30:393-409. 25851843 10.1177/0269215515579286

